# Nephrotic Syndrome Secondary to Proliferative Glomerulonephritis with Monoclonal Immunoglobulin Deposits of Lambda Light Chain

**DOI:** 10.1155/2014/164694

**Published:** 2014-07-22

**Authors:** Seongseok Yun, Beth L. Braunhut, Courtney N. Walker, Waheed Bhati, Amy N. Sussman, Faiz Anwer

**Affiliations:** ^1^Department of Medicine, University of Arizona, Tucson, AZ 85721, USA; ^2^Department of Medicine, Arizona Health Sciences Center, 6th Floor, Room 6336, 1501 N. Campbell Avenue,Tucson, AZ 85719, USA; ^3^Division of Pathology, University of Arizona, Tucson, AZ 85721, USA; ^4^Division of Hematology, Oncology, Blood & Marrow Transplantation, Department of Medicine, University of Arizona, Tucson, AZ 86721, USA

## Abstract

We describe a rare case of a 46-year-old woman with history of refractory nephrotic syndrome and hypertension who presented with worsening proteinuria and kidney function. Work-up for both autoimmune and infectious diseases and hematologic malignancies including multiple myeloma were negative. Kidney biopsy demonstrated glomerular sclerotic change with lambda light chain deposits in the subendothelial space, which is consistent with proliferative glomerulonephritis with monoclonal immunoglobulin deposit (PGNMID). The patient was treated with bortezomib and dexamethasone without clinical improvement and eventually became hemodialysis dependent.

## 1. Introduction

PGNMID is a recently described as a rare disorder that belongs to the class of disorders known as monoclonal gammopathy of renal significance (MGRS) [1]. PGNMID has been shown to be associated with various underlying diseases including myeloma, chronic lymphocytic leukemia (CLL), and parvovirus infection [1–5], and the most common clinical manifestation is renal involvement. PGNMID has unique histological and pathophysiological features, which distinguish it from other entities of MGRS or malignant monoclonal diseases. The renal deposit in PGNMID is composed of monoclonal immunoglobulin (Ig). Microscopic findings typically show a nonfibrillar or nonmicrotubular pattern of Ig deposit without any organized structure. Although histologic features have been well described and the clinical course of PGNMID is known to be poor, the underlying mechanisms of PGNMID remain elusive. Based on the evidence of other clonal disorders, bortezomib, cyclophosphamide, and high dose of chemotherapy followed by stem cell transplant are the current recommended treatments, although with variable response.

## 2. Case Presentation

**Figure 1:**
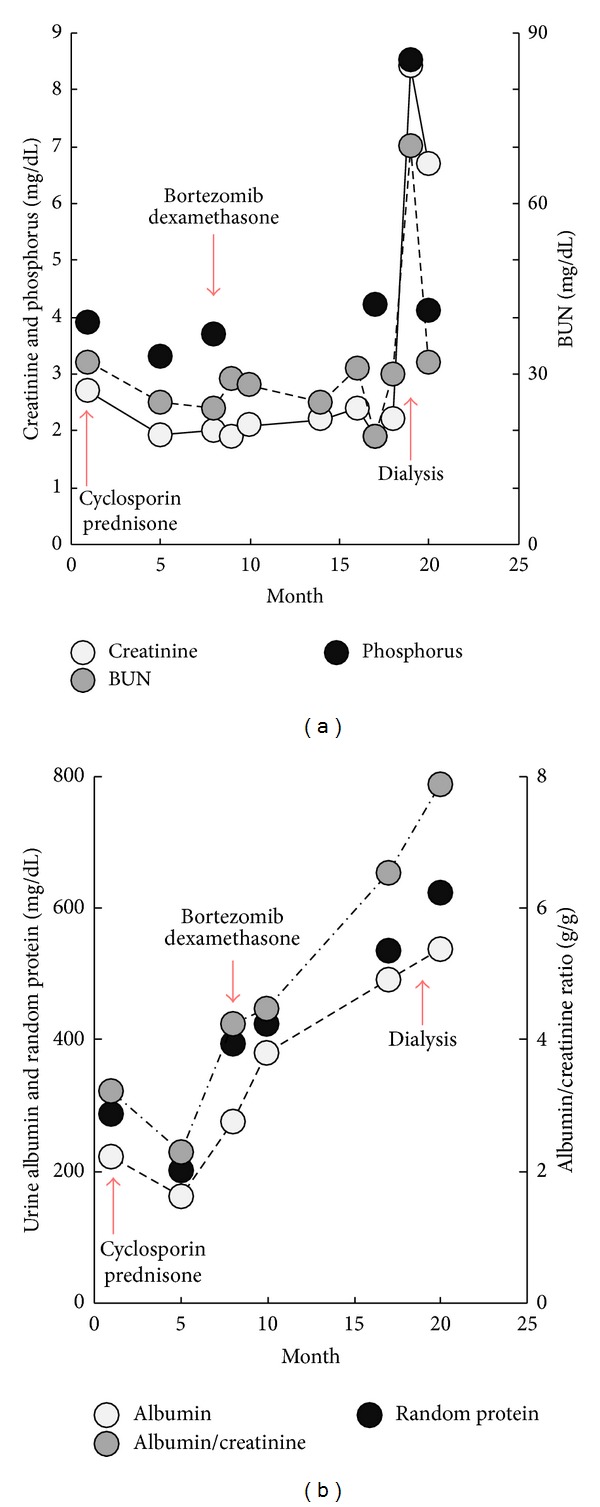
Laboratory values during the chemotherapy. 16-week treatment with cyclosporine followed by prednisone failed to prevent disease progression. Patient received a total of 4 cycles of bortezomib and dexamethasone, however, with no clinical or laboratory improvement. Disease progressed and patient eventually became hemodialysis dependent.

A 46-year-old woman with history of nephrotic syndrome was referred from an outside hospital for further evaluation and management of recurrent and progressively worsening nephrotic syndrome with stage III chronic kidney disease (CKD). Kidney biopsy and immunofluorescence (IF) staining one year ago had shown focal segmental proliferative glomerulonephritis and the patient had been treated with 16 weeks of cyclosporine followed by prednisone. Unfortunately, one year later, she presented with recurrent nephrotic symptoms including elevated serum creatinine, hypoalbuminemia, and significant proteinuria (Figure 1).

**Figure 2:**
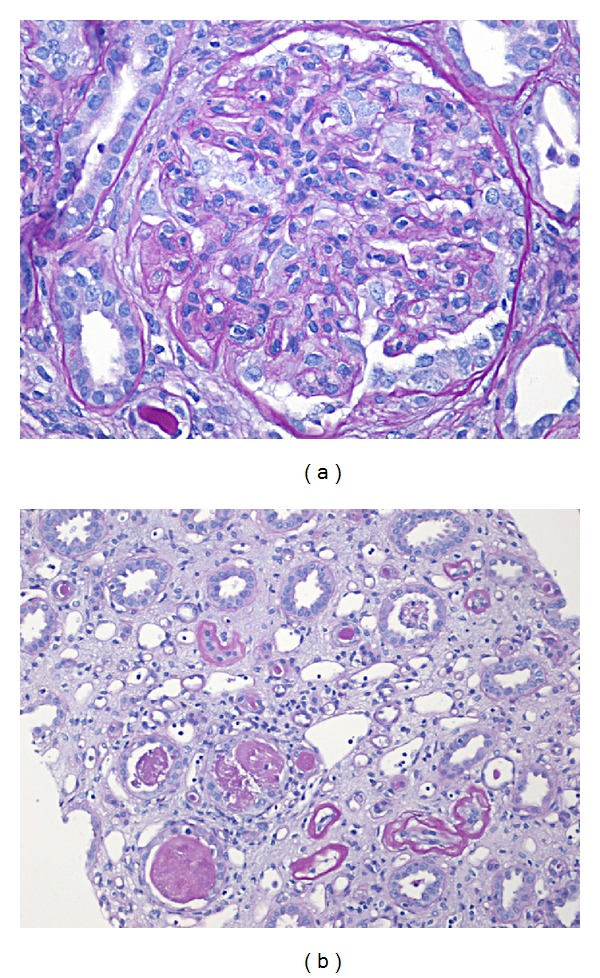
Light microscopic finding of kidney biopsy. PAS at 400x shows glomerulus with globally increased mesangial matrix and cellularity and segmental attachment to Bowman's capsule (a). The glomerular capillary loops show patchy endocapillary proliferation and focal mononuclear cell infiltration. No necrosis or crescents are seen in glomeruli, and the interstitium shows extensive fibrosis and patchy tubular atrophy. PAS at 200x demonstrates tubules containing intraluminal casts with smooth borders and flattening and sloughing of tubular epithelial cells (b).

**Figure 3:**
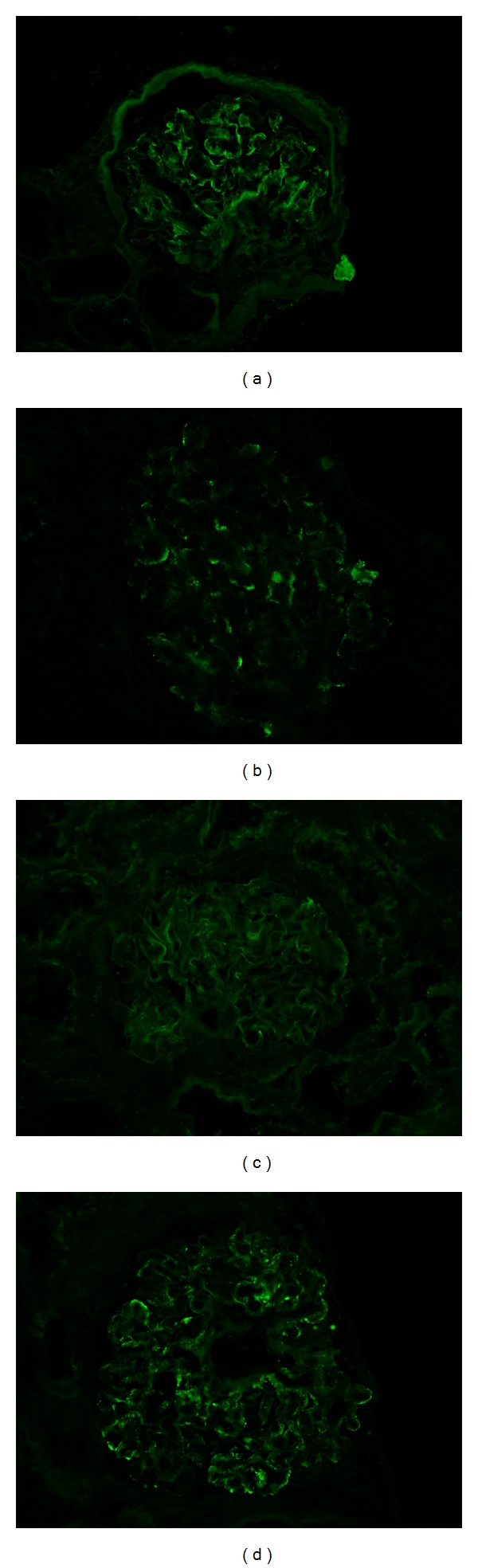
Immunofluorescence staining of kidney biopsy. Immunofluorescence staining shows trace mesangial staining for IgG (a), IgM (b), and kappa light chain (c) but 2+ mesangial and granular capillary loop staining for lambda light chain (d), confirming the diagnosis of PGNMID with monoclonal lambda light chain.

**Figure 4:**
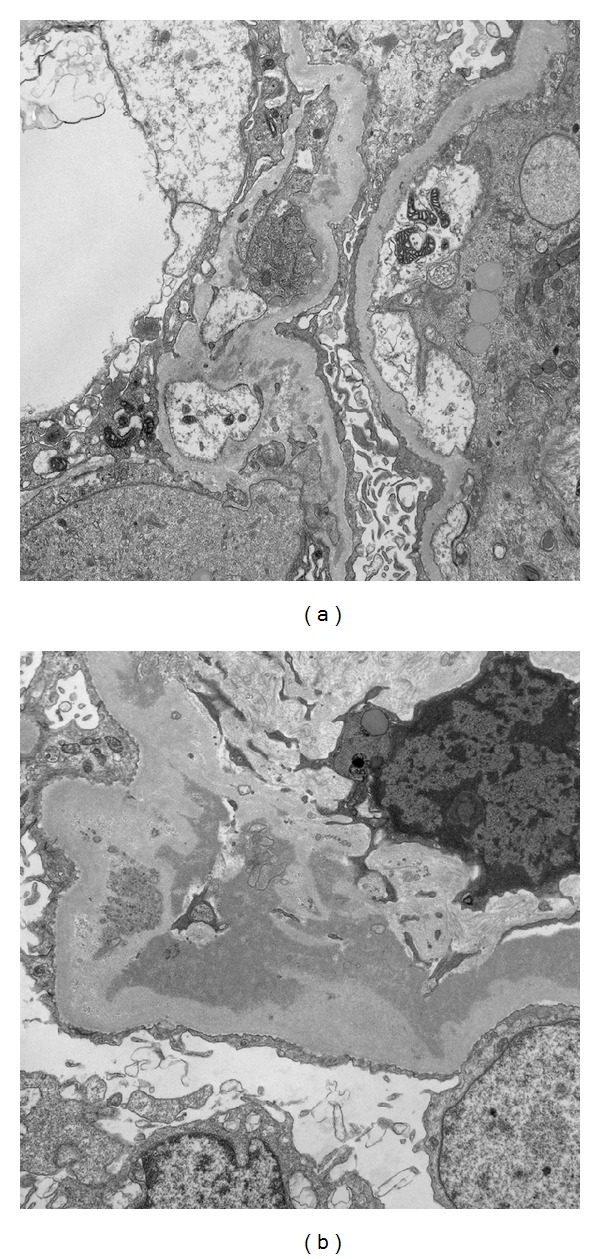
Electron microscopic finding of kidney biopsy. Electron microscopy demonstrates diffuse podocyte foot process effacement, proliferation in the capillary loop, and remodeling of the glomerular basement membrane with cellular interposition (a). The glomerular basement membrane is markedly irregular in thickness, and dense deposits are located in the mesangial areas and focally within subendothelial space without any organized substructural features (b). In loops without these abnormalities, the glomerular basement membrane is of normal thickness and has normal architecture.

Vital signs revealed blood pressure of 155/83 mmHg, heart rate of 81 beats per minute, respiratory rate of 14/minute, and temperature of 37.6°C. Lung and heart exams were normal and there was no palpable cervical, supraclavicular, axillary, or inguinal lymphadenopathy. Bilateral, one plus pitting lower extremity edema was present. Laboratory exam showed serum creatinine of 1.92 (normal 0.67–1.17 mg/dL) (Figure 1), IgG of 1333 (normal 552–1631 mg/dL), elevated kappa chain of 3.67 (normal 0.33–1.94 mg/dL), and lambda chain of 2.29 (normal 0.57–2.63 mg/dL) with normal ratio of 1.62. A 24-hour urine protein excretion was 8 g (normal 80 mg), and urine and serum immunofixation demonstrated positive monoclonal-free lambda light chain. Serum IgA (201 mg/dL), IgM (102 mg/dL), C3 (152 mg/dL), and C4 (32 mg/dL) levels were within normal range. Additional rheumatologic work-up included anti-SSA, anti-SSB, anti-nuclear antibodies (ANA), anti-neutrophil cytoplasmic antibody (ANCA), serum cryoglobulin, hepatitis panel, and serum protein electrophoresis (SPEP), which were all negative. Renal biopsy with light microscopic examination revealed multiple globally sclerosed glomeruli and occasional glomeruli with segmental sclerosis. The glomerular capillary loops showed patchy endocapillary proliferation and mononuclear inflammatory cell infiltration. A patchy mononuclear cell infiltrate was observed with associated tubulitis (Figures 2(a) and 2(b)). Immunofluorescence (IF) examination revealed mesangial staining for IgG (trace) and IgM (trace) and glomerular capillary loops were stained positive for C3 (1 to 2+) and lambda light chain (2+) (Figures 3(a)–3(d)). Subsequent electron microscopic ultrastructural examination demonstrated scattered electron dense immune-type deposits within mesangial areas and focally within the subendothelial space. The complexes did not show any organized substructural features, and the glomerular basement membrane had markedly irregular thickness in areas involved with deposits and/or endocapillary proliferation (Figures 4(a) and 4(b)). Further work-up for multiple myeloma, including skeletal survey and bone marrow biopsy, was normal with only 5% plasma cells on bone marrow immunohistochemistry and only 0.6% CD20+ B cells on flow cytometry. Additional fluorescence in situ hybridization (FISH) analysis included 1q21 (CKS1B), 9q34 (ASS1), 11q13 (CCND1), 14q32 (IGH), 15q22 (PML), and 17p13 (TP53), which were all negative. Although the IF staining for IgG is weaker than that of previous case reports, suggesting either early stage of disease or suboptimal staining condition, the microscopic findings and laboratory results confirmed the diagnosis of proliferative glomerulonephritis with monoclonal immunoglobulin deposit (PGNMID) with monoclonal lambda light chain.

The patient completed three cycles of weekly bortezomib along with dexamethasone without complication. Unfortunately, her disease progressed and she became hemodialysis dependent (Figure 1).

## 3. Discussion

PGNMID is a rare disease (incidence of 0.17%) with most published data extrapolated from case reports [1]. Proposed criteria to diagnosis PGNMID include (1) the presence of Ig deposit with a single IgG subclass and a single light chain isotype, (2) granular pattern of deposit under microscopy, and (3) no evidence of cryoglobulinemia [6]. PGNMID belongs to the disease spectrum known as monoclonal gammopathy of renal significance (MGRS) that includes amyloidosis (AL), type I cryoglobulinemia, immunotactoid glomerulopathy (ITG), and Randall-type monoclonal immunoglobulin deposition disease (MIDD) [7]. AL is one of the most frequent diseases in MGRS and is associated with plasma cell clones [6]. The deposit is composed of monoclonal light chain or its fragments. Although 70–80% of AL patients have renal involvement, the mortality largely depends on cardiac involvement. Type I cryoglobulinemia is another monoclonal disease with single monoclonal Ig deposit [6]. IgG type is the most common type and is precipitated by cold temperature. Raynaud's phenomenon, glomerular disease of membranoproliferative glomerulonephritis (MPGN), hypertension, and articular involvement are common clinical manifestations (Table 1). Randal-type MIDD is frequently associated with multiple myeloma with more than 10% of bone marrow involvement with plasma cells [6]. The most common form of Randal-type MIDD is light chain deposition disease (LCDD). Light heavy chain deposition disease (LHCDD) and heavy chain deposition disease (HCDD) are relatively rare. Glomerular deposits with nodular glomerulosclerosis and linear amorphous deposits in the tubular basement membranes are common histologic findings. Majority of the patients present with renal involvement including proteinuria, hematuria, and renal insufficiency. Renal biopsy and pathologic review are mandatory for the differential diagnosis of MGRS.

B-cell clones that do not meet criteria for lymphoma or multiple myeloma are known to be responsible for MGRS. Most of the pathological and clinical consequences of MGRS arise from the deposition of monoclonal Ig in the organ rather than the proliferation of abnormal B-cell clones [8]. Moreover, PGNMID has a distinct microscopic appearance compared to other entities of MGRS, including a granular nonlinear pattern without deposits in glomerular or tubular basement membranes [1, 2, 6] and diffuse endocapillary proliferative glomerulonephritis (DPGN) or membranoproliferative glomerulonephritis (MPGN) pattern.

Patients with PGNMID most commonly present with proteinuria, renal insufficiency, and hematuria without clinical evidence of multiple myeloma or B-cell lymphoproliferative disorders such as lymphadenopathy, hepatosplenomegaly, or skeletal involvement, and extrarenal manifestations of PGNMID are extremely rare (Table 2). Additionally, the renal Ig deposits are not a consequence of glomerular damage secondary to autoimmune, infectious, or systemic disorders that can cause antigenic stimulation. Overall, the pathogenesis of PGNMID is poorly understood. Only one-third of patients showed a positive monoclonal spike in either SPEP or UPEP, and the plasma cells found in the bone marrow are usually less than 10% [1]. In our patient, serum kappa light chain was slightly elevated and lambda chain was normal with normal ratio of 1.62. However, monoclonal renal deposits indicate the presence of circulating monoclonal plasma cells and an underlying B-cell disorder as shown in our patient, and this has been the rationale for using chemotherapy targeting B-cells in PGNMID [9].

To date, there is no treatment that inhibits the process of Ig accumulation or removes already formed immune deposits. Currently, most therapies for PGNMID target the underlying B-cell disorder using chemotherapeutic or immune-modulatory agents, although evidence is lacking. There are several case reports of PGNMID patients treated with chlorambucil, mycophenolate mofetil, prednisone, thalidomide, and rituximab, but the outcomes are disappointing with low remission rates [1, 4, 10]. The International Kidney and Monoclonal Gammopathy Research Group recommends treatment according to the severity of renal disease (Table 2) [9]. Symptomatic management is recommended for CKD stage I or II with proteinuria less than 1 g/day and no evidence of disease progression. Chemotherapy with bortezomib or cyclophosphamide is recommended for CKD stage I or II with proteinuria greater than 1 g/day and progressive disease. High dose melphalan followed by autologous stem cell transplant (HDM/ASCT) is an alternative option for patients less than 65 years of age. However, the efficacy of these chemotherapy targeting plasma cells is unknown especially in a setting of less than 10% plasma cells in PGNMID warranting further clinical investigation. Accordingly, rituximab targeting CD20+ B cell clones is excluded from current recommendations due to its limited efficacy and delayed complications [11], although it has been shown to be effective in several PGNMID cases [4]. Lastly, kidney transplantation with or without pretransplant chemotherapy or HDM/ASCT is the best suggested option for patients with CKD stage V.

Following the recommendation of the International Kidney and Monoclonal Gammopathy Research Group, our patient was treated with bortezomib and dexamethasone, although without improvement. Rituximab was not considered for this patient due to lack of CD20+ population in flow cytometry in bone marrow cells. Eventually, the patient's renal disease progressed to hemodialysis dependence. Alternative treatment options are now HDM/ASCT or kidney transplant.

As described in this case, there are several limitations in the current treatment guidelines. First of all, therapies are not based on prospective, randomized controlled clinical trials but on a limited number of case studies. Secondly, treatment simply depends on CKD staging without an understanding of the underlying pathophysiology of monoclonal production and deposition. Thirdly, no prognostic factor or prognostic system has been investigated or developed yet. Finally, more clinical studies are necessary to better understand PGNMID and improve clinical outcomes of this rare disease.

## 4. Conclusion

PGNMID is a very rare disease of the MGRS spectrum. The pathologic abnormalities are limited to the renal system, and common clinical manifestations include proteinuria, hypoalbuminemia, and renal insufficiency. The underlying mechanisms of PGNMID remain elusive. However, the presence of monoclonal deposits in the kidney suggests an underlying plasma cell and B-cell disorder. Depending on the stage of CKD, the current therapeutic options are bortezomib, cyclophosphamide, hemodialysis, or stem cell transplant, although evidence is lacking.

## Figures and Tables

**Table 1:** Clinical manifestations of PGNMID [1].

Pathologic findings	Clinical findings	Laboratory findings
Membranoproliferative GN	Peripheral edema	Proteinuria
Endocapillary proliferative GN	Hematuria	Hypoalbuminemia
Mesangial proliferative GN		Renal insufficiency
Membranous GN		Serum and urine paraprotein
Focal interstitial inflammation		Low C3 and C4
Tubular atrophy and interstitial fibrosis		

**Table 2:** Treatment recommendation for PGNMID [9].

CKD stage I and II Proteinuria (<1 g/day) No evidence of progressive disease	CKD stage I and II with high grade proteinuria (>1 g/day) Progressive disease CKD stage III and IV	CKD stage V
(1) Symptomatic measures only (2) Careful surveillance	(1) Chemotherapy, cyclophosphamide, and bortezomib (2) HDM/ASCT (3) Rituximab	(1) Kidney transplantation (2) HDM/ASCT
